# Attitudes and Practices Regarding Research among Romanian Medical Undergraduate Students

**DOI:** 10.3390/ijerph19031872

**Published:** 2022-02-08

**Authors:** Andreea Iulia Pop, Lucia Maria Lotrean, Anca Dana Buzoianu, Soimita Mihaela Suciu, Mira Florea

**Affiliations:** 1 Department of Community Medicine, Iuliu Hatieganu University of Medicine and Pharmacy, 400012 Cluj-Napoca, Romania; pop.andreea.iulia@elearn.umfcluj.ro (A.I.P.); miraflorea@umfcluj.ro (M.F.); 2 Department of Functional Biosciences, Iuliu Hatieganu University of Medicine and Pharmacy, 400012 Cluj-Napoca, Romania; abuzoianu@umfcluj.ro (A.D.B.); mihaela.suciu@umfcluj.ro (S.M.S.)

**Keywords:** research, medical undergraduate students, Romania

## Abstract

Objectives: This study focuses on the assessment of attitudes and practices regarding research among undergraduate medical students from Cluj-Napoca, Romania. Material and Methods: A cross-sectional study was performed thorough anonymous questionnaires (May–June 2018) among 510 third- and fifth-year students of Iuliu Hatieganu University of Medicine and Pharmacy from Cluj-Napoca, Romania. Results: More than 60% of the third- and fifth-year students declared that they were interested and willing to perform research during medical studies, while more than two-thirds were interested in doing so after graduation. In total, 6% of third-year students and 31% of fifth-year students declared that they had prepared a scientific presentation for a medical congress at least once. Around 9% of the third-year students contributed to the writing of a scientific article and participated in research projects. Among fifth-year students, one-quarter were involved in writing scientific papers, and 21% participated in research projects. Conclusions: To the best of our knowledge, this study assesses, for the first time in Romania, the perspectives and behaviors of medical undergraduate students with regard to involvement in research activities. The results show that Romanian medical students value opportunities for conducting research, which encourages institutional initiatives that support their involvement in curricular and extracurricular research activities.

## 1. Introduction

In contemporary medical university education, the importance of training, exposure, and involvement of students in research activities is emphasized [1,2,3]. Several studies and organizations have underlined different benefits derived from integrating research into the medical education of undergraduate students [1,2,4,5].

First, it is a valuable means for training medical students to practice in an increasingly complex society, with a strong need for evidence-based medicine which requires medical doctors to make informed medical judgements based on the best available evidence. Hence, even if they are not actively engaged in research themselves, medical doctors need to understand how evidence is derived, and should, at least, have basic knowledge and skills to understand research processes, be capable of making critical appraisal of research results, and put their findings into practice [4,5,6]. During undergraduate medical education, research instruction and involvement provides, probably, the first opportunity and, for some of the future physicians, one of the few real opportunities which contribute to the development of their capacity to understand scientific methods applied to medical practice, which will stimulate critical thinking [5,6,7].

On the other hand, several studies have shown that performing undergraduate research, whether organized or extracurricular, stimulates a positive attitude in students toward research and supports their involvement in research activities later in their career [5,6,7,8]. Moreover, medical students can play a role in the research productivity of research groups from the university [9,10,11].

The training of medical students and their involvement in curricular and extracurricular activities related to research varies between countries and universities [5,12,13,14,15,16,17]. However, to the best of our knowledge, there are no published studies which investigated the opinions and behaviors regarding involvement in research activities among medical undergraduate students from Romania.

This study focuses on assessing the attitudes and practices regarding research among undergraduate medical students from the Faculty of Medicine of Iuliu Hatieganu University of Medicine and Pharmacy from Cluj-Napoca, Romania, one of the main medical faculties in Romania, situated in the north-west part of the country.

The undergraduate medical studies at the Faculty of Medicine from Cluj-Napoca include 6 years. In the second year of their course, the students of the Faculty of Medicine from Cluj-Napoca have a module regarding the methodology of scientific research, which aims to teach students how to critically analyze medical research (21 h of lectures and 21 h of labs). The graduation exam at the end of the medical studies course includes a component for the preparation and defense of a graduation research thesis—an original scientific paper, written according to rigorous principles under the coordination of a faculty teaching staff. Students are required decide the topic and coordinator of the graduation thesis during the fifth year of studies at the latest [18]. At the same time, undergraduate students also have possibilities to be engaged in extracurricular research activities organized by the faculty teaching staff from various departments. This involvement is subject to the personal preferences of students and the availability of teachers, and is not monitored or coordinated at institutional level; no information is available currently with regard to awareness, motivation, behavior, and intention of students to take advantage the of available opportunities.

Hence, this study has three main objectives. The first is to assess attitudes regarding involvement in research as well as their interrelationship among Romanian medical undergraduate students from the Faculty of Medicine from Cluj-Napoca, Romania. The second is to obtain information about research activities already performed by the participating students, as well as the factors associated with these behaviors. Last, but not least, we aim to assess the willingness for involvement in research activities during and after their medical studies and the factors which influence this interest.

## 2. Materials and Methods

### 2.1. Study Sample and Data Collection

A cross-sectional study was performed during May–June 2018 among third- and fifth-year students of the Faculty of Medicine from the Iuliu Hațieganu University of Medicine and Pharmacy from Cluj-Napoca, Romania. The study received the approval of Ethic Commission of Iuliu Hatieganu University of Medicine and Pharmacy (Approval no. 194/19 April 2018).

All students in years 3 and 5 from the Romanian section of the faculty were invited to participate in the study by completing a paper and pencil anonymous questionnaire that was distributed and collected by the members of the research team at the end of the teaching activities in which the students participated in different departments between May and June 2018.

The final sample included 510 participants, of which 260 were students in year 3 of study, out of a total of 324, and 250 were students in year 5 of study, out of a total of 308. Thus, the participation rate of students in year 3 was 80.2%, and for year 5 the rate was 81.1%. Among the respondents of year 3, 36.5% are male and 63.1% are female. Among the respondents of year 5, 29.6% are male, and 70.4% are female, in accordance with the fact that a higher percentage of women study at the Faculty of Medicine from Cluj-Napoca than men.

### 2.2. Instrument for Data Collection

The questionnaire was developed for this study and assessed socio-demographic characteristics (age, gender), as well as several opinions and behavior of undergraduate medical students regarding research. The investigated opinions were related to the following issues:

- Research is important for professional development (Possibilities of answers on a 5-point scale ranging from ’I totally agree’ to ‘I totally disagree’);
- Factors which influence the involvement of undergraduate students in research activities: individual factors (personal motivation and interest, the desire to improve the personal curriculum vitae), social influences (the influence coming from other colleagues), professional influences (presentation by the teaching staff regarding the research opportunities for students, the help and mentoring from the teaching staff), and institutional factors (research training for undergraduate students, the existence of research grants for undergraduate students); the options for answers were on a 5-point scale ranging from ‘To a significant extent’ to ‘Not at all’.

At the same time, the questionnaire investigated if during the undergraduate studies the students were involved in some form of research as part of the graduation thesis or more complex research activities). The questionnaire also asked if students had participated in a medical congress, if they had presented papers at medical congresses (oral or poster presentations), if they had participated in writing a scientific article, and if they participated in research projects. Moreover, the questionnaire tried to obtain information regarding motivation and willingness to become involved in research activities during and after medical studies (Possibilities of answers: ‘Yes’, ‘No’, ‘I do not know’).

### 2.3. Data Analyses

The prevalence and mean for the investigated issues were calculated, while chi^2^ tests and *t*-tests were used in order to assess differences between students from the third- and fifth-year of study.

Bivariate correlation was employed to assess interrelationship between several attitudes regarding involvement in research of medical students. According to Cohen, strong correlation was considered at *r* > 0.50, while medium and small correlation was considered at *r* > 0.30 and *r* > 0.10, respectively.

An index for involvement of medical students in research (research index) was created by summing the involvement in the following research activities: participation at medical congresses (0–no, 1–yes), presenting papers at medical congresses (oral or poster presentations; 0–no, 1–yes) participation in writing a scientific article (0–no, 1–yes), and in research projects (0–no, 1–yes); the minimum value was 0, the maximum 4. Stepwise multivariate linear regression analyses (using forward selection) were used to assess the factors associated with higher involvement in research activities (measured as higher research index). The independent variables were gender (coded 0–males, 1–females), opinions about the importance of research and factors which influence the involvement of students in research; the analyses were performed separately for the third- and fifth-year students.

Stepwise multivariate logistic regression analyses (using forward selection) were employed for a better understanding of factors which influence the interest and willingness of undergraduate students in research during undergraduate studies as well as after graduation, and the different factors (gender, opinions about the importance of research and factors which influence the involvement of students in research, previous involvement in different forms of research). The analyses were performed separately for the third- and fifth-year students.

Statistical analyses were performed using IBM SPSS Statistics for Windows, Version 20.0. (Armonk, NY, USA: IBM Corp.), statistical significance being considered at *p* < 0.05.

## 3. Results

### 3.1. Attitudes Regarding Research and Their Relationship

The results show that the majority of students from both years of study recognize the importance of research for the medical profession, with one-third of the students from the third year and almost half of those from the fifth year being in complete agreement with this. Students from the fifth year had a stronger recognition of the importance of research for the medical profession (See Table 1).

When asked about the factors which stimulate the involvement of medical students in research activities, the students from the third year recognized personal motivation and interest, as well as mentoring and help from the university teaching staff as the main factors (around 60% and 48% of students, respectively, believe these factors contribute to involvement in research activities to a very high extent). Around 40% of the third-year medical students agree that the promotion of the research opportunities by university teaching staff and the opportunities to access grants increase student involvement to a very high extent, while almost one-third consider that the intention to improve CVs and the opportunity for training in the field of research field also contribute to a high extent. The influence/example of colleagues seems less important, but still half of them believe that this factor contributes to a high extent.

Among the fifth-year students, personal motivation and interest, mentoring and help from university teaching staff, as well as the possibility to access grants were the main factors; around half of the students believed they contributed to a very high extent to motivating students to participate in research activities. Appropriate training and presentation from the university teaching staff were recognized as factors contributing to a very high extent by 46% and 42% of students from the fifth year, respectively. The influence of colleagues was considered less important, but around half of them consider it important to a high or very high extent.

Comparing the two groups, it is noticed that the fifth-year students to a greater extent recognized the importance of research training and the possibility to access research grants.

Table 2 shows that there were low to medium, and even high correlations between all the investigated attitudes regarding research. The opinion that research is important for professional development had the strongest correlation with attitudes that research involvement is stimulated by personal motivation and interest, but also by presentations from the teaching staff.

Individual factors influencing research involvement had low to moderate correlation with each other and with the other factors. Those who think personal motivation and interest are important are also more prone to think that help and mentoring from the teaching staff and research training for students are important. On the other hand, students’ desire for improving their CV through research had the strongest association with social influences coming from colleagues and recognizing the importance of receiving research grants for students. Recognizing the social influences from colleagues is also moderately correlated with recognizing the importance of professional influences.

It is also noticed that there is a strong association between the two dimensions of the assessed professional influences (presentations from the teaching staff and help and mentoring from the teaching staff), a moderate to high association between the two dimensions of assessed institutional factors (research grants for students and research training for students), as well as between the professional influences and institutional factors.

### 3.2. Practices Regarding Research and Factors Associated with Them

Table 3 shows that 10% of the third-year group declared that they were involved in some forms of research activities already (1.2% regarding their graduating thesis and the rest other types), while almost half of the fifth-year did so (28% with regard to graduating thesis and the rest other, more complex research activities).

The majority of students participated in medical congresses, but 6% of the third-year and 31% of the fifth-year students declared they had made a scientific presentation to medical congresses at least once. Around 9% of the third-year students contributed to writing a scientific article and participated in research projects. Among fifth-year students, one-quarter were involved in writing scientific papers and 21% participated in research projects.

With regard to the research index, it was 0 for 16.5% of the third-year students and 10.8% of the fifth-year students. Around two-thirds and half, respectively, of the two groups of students had the value of the index 1, while near 5% of the third-year students and one out of five students from the fifth-year had the value of the index 3 or 4 (see Table 3). A statistically significant difference between the two groups was noticed, the fifth-year students having a higher research index.

As presented in Table 4, the results of multivariate linear regression analyses show that, among both third-year students and fifth-year students, the research index was higher among those who were more convinced that research is important for professional development. At the same time, among the fifth-year students, the research index was higher among males than females, and among those being more convinced that personal motivation and interest are influencing the involvement in research.

### 3.3. Willingness to Become Involved in Research Activities in the Future and Associated Factors

Furthermore, when asked about their motivation and willingness to become involved in the medical studies after graduating, among third-year students, two-thirds said ‘yes’, one-quarter were undecided, and 6% declared ‘no’. Among fifth-year students 74% are willing, one out of five was not decided, and 6% said no.

As presented in Table 5, the results of the multivariate logistic regression analyses show that, among third-year medical students, the willingness to become involved in research during medical education was higher among those who were more convinced about the importance of research for professional development, as well as about the fact that presentations from the teaching staff influence students’ involvement in research. Among fifth-year medical students, the factors associated with higher willingness were having stronger beliefs that research is important and being more convinced that the possibility to access grants stimulates the motivation of students, as well as having previous engagements in research projects.

With regard to the willingness to engage in research after medical graduation, the results of the multivariate logistic regression analyses show that the associated factors for both years are the importance given to research, considering that the professional influences are important (presentation from teaching staff regarding available research opportunities for third-year students and help and mentoring offered by teaching staff for fifth-year students) and the previous participation in medical congresses of undergraduate students (see Table 6).

## 4. Discussion

This study assesses, for the first time in Romania, the perspectives and behaviors of medical undergraduate students with regard to involvement in research activities, the target group being third- and fifth-year students from the Faculty of Medicine from Cluj-Napoca, Romania.

Different studies from other countries underline the current emphasis on research in medical education which more broadly reflects the preoccupations in higher education, with several universities stimulating the research activities in different ways and at different levels [19,20,21,22,23]. In the Faculty of Medicine from Cluj-Napoca, research is a priority, despite limited funding for research from national sources. The undergraduate medical students have a mandatory module regarding the methodology of scientific research in the second year of study; for more than three decades it has been mandatory for the students to prepare and submit a graduation thesis performed under the supervision of a member of the faculty teaching staff in order to graduate. Students are encouraged to choose their supervisor and the theme of the graduation thesis in the fifth year, while by the end of the sixth year the thesis must be finished. It contains two parts (one presenting data from literature with regard to the selected subject, one presenting personal results with regard to the selected subject). The thesis is presented in front of a commission and the mark represents an important part of the graduation mark, in addition to the theoretical and clinical examination. Hence, students have the opportunity to improve skills regarding critical review of literature, development of study objectives and methodology, data collection, analyses, interpretation, and presentation in the forms of reports and oral presentations.

A recent review examining the perceptions of medical students regarding research during undergraduate medical school showed that, worldwide, medical students recognize the necessity and importance of research in medical education, as reflected by many students reporting positive attitudes and interest in research endeavors [5]. The data from our study also confirms that the majority of students consider research as being important.

Despite the varied approaches and efforts at improving research during medical school, providing opportunities for the development of effective and diverse research skills within undergraduate medical education is facing several challenges and difficulties [2,3,5]. Studies performed in other countries underline that there are both institutional and non-institutional factors which influence successful research participation of undergraduate medical students [7,12,17,24]. Our study investigated the opinions of Romanian medical students regarding the factors which could influence the involvement of medical students in research, the focus being on individual factors (personal motivation and interest, the desire to improve their CV), social influences (the example of other colleagues), professional influences (presentations of research opportunities from teaching staff, mentoring and help from the teaching staff), and institutional factors (offering research training, research grants for students).

Both third-year and fifth-year students scored the role of personal motivation and interest and the mentoring and help from the teaching staff highest of all factors. The other professional influences and institutional factors also received high scores, with students from the fifth-year appreciating the role of research training and access to grants more, probably as a consequence of the fact that, being more involved in research activities and research projects, they understand better the process of research from the idea to data gathering and dissemination of results. Studies from other countries also underline the fact that the support offered by teachers and being aware of research activities performed at one’s own university and the opportunity to participate, as well as appropriate training and access to infrastructure and funding, are considered important factors by students in stimulating participation in research during medical studies [7,12,17,24]. Moreover, our study shows a clear association between personal motivation and interest and professional influences and institutional factors, the strongest association being with mentoring and help from the teaching staff and the offer of research training by the faculty. Since all students from the Faculty of Medicine from Cluj-Napoca start to have discussions and collaboration with a teacher in order to decide and formulate the subject and the methodology for their graduation thesis by the end of the fifth year, at the latest, they realize that the help offered by the teachers can stimulate their interest in performing research, which might lead to a deeper and more complex research activity than just the graduation thesis. Due to the cross-sectional design of this study, we cannot identify causality, but the relationship can be in both directions, students who have interest and motivation in performing research realize the importance of finding a good research mentor, as well as research training which will help them to develop appropriate skills.

At the same time, there was a strong correlation between the two professional influences, showing that there are students who realize that it is important to find a good mentor, while the presentations performed by the teachers could help them to better understand the research opportunities and the research subjects/teams which could offer them help for their graduation thesis and beyond. There were also moderate to high correlations between opinions that professional influences coming from teachers and institutional factors related to offering opportunities for training and funding research are important, showing that there are several students who understand the need for institutional support for students, as well as for teachers, in order to build an environment which provides tools and motivation for enhancing research among students as a part of the research plan of the faculty. Older students, already having more experience with research activities, are more aware of the importance of institutional support through research training and the possibility to access research grants for students.

In our study, the desire to improve their curriculum vitae and the example of colleagues was scored lower by the students, this also comes as a consequence of the fact that, in the Romanian medical system, training opportunities and jobs after graduation are obtained based on exams which test medical knowledge, and not so much on the basis of a CV. Nevertheless, there was a moderate correlation between these two factors, showing that the example of other colleagues might lead to the desire to perform research in order to have a CV as good as other peers, which might also lead to several opportunities in the future.

With regard to practices, our study shows that only 10% of the third-year students were involved in research activities, but almost half of the fifth-year students declared they were involved in different forms of research (25% only in research regarding graduation thesis and 20% even in more complex research). Moreover, one out of ten third-year university students and one out of five students from the fifth year declared involvement in research projects.

The knowledge generated by research should be disseminated through presentation at scientific meetings and the publication of scientific papers; the involvement of students in these processes being important for development of specific skills, making them understand the importance of their work, stimulating them to increase the quality of their work in order to generate results which are valuable, and succeeding in becoming published, as well as to increase the visibility and productivity of several research groups from the faculty [9,10,11,25]. Our results show that less than 10% of the third-year students presented at medical congresses or were authors of scientific papers sent for publication in scientific journals, while 31% of the fifth-year students presented at medical congresses and one-quarter were contributing for a scientific article. Few studies have looked at the number of papers published or presented by medical students at scientific meetings worldwide [2,9,10,11]. For instance, an article published 10 years ago investigated publication practices of medical students at British medical schools and showed that 14% had submitted an article for publication while at medical school, while 17% presented a paper (oral presentation or poster) during scientific meetings, with students from the final years of study being more involved in these types of activities [2]. There are also studies which show that students who are involved in mandatory or voluntary research programs increase their chances of publishing or presenting scientific results [5,24]. On the other hand, in our study, more than 80% of the students from both university years declared they attended medical scientific meetings, but without to make a presentation, showing that there is interest among students for this type of activity.

The calculated research index (minimum 0, maximum 4) was lower than three for the majority of students, while around 5% obtained the maximum score, with students from the fifth year having higher scores and males from the fifth year having stronger scores than females from the same year. The research index included research activities (participating to medical congresses, presenting papers at medical congresses, being part of research projects, becoming involved in writing research papers) which are not mandatory for the graduation thesis and to some extant could be implied through the end of the graduation thesis, when the results and their interpretation are clear. Nevertheless, there were students who were involved in these types of activities even before the final year of studies, in strong relationship with their opinion that research is important for professional development, as well as being convinced that personal motivation and interest is an important ingredient for stimulating students’ involvement in research.

At the same time, more than 60% of the third- and fifth-year students declared they are interested and willing to perform research during medical studies, while more than two-thirds of both groups have the interest and willingness to perform some forms of research after graduation.

Interest and willingness to perform research, both during undergraduate studies and after graduation, was higher for third-year students among those who recognized better the importance of research and were more convinced about the importance of presentations made by teachers with regard to research opportunities for students, while the research after graduation was also desired more by students who participated in medical scientific meetings. Among the fifth-year students, beside a better recognition of the importance of research, previous involvement in research projects and better understanding of the role of funding increases the willingness to perform research during undergraduate studies. Fifth-year students who recognize the importance of research, have stronger attitudes regarding the role of mentoring and help coming from teaching staff also declared a higher interest in performing research after graduation. All these prove, once again, the importance played not only by individual factors, but also by professional influences and institutional factors, as well as by early exposure to different forms of research activities in shaping the interest of undergraduate students in becoming involved with research during and after graduation.

This study is subject to several limitations. It is a cross-sectional study, so causality with regard to relationship between attitudes and practices cannot be established. At the same time, it included only third- and fifth-year students and used an exploratory questionnaire to assess opinions and behaviors regarding research among undergraduate medical students. Future studies involving both quantitative and qualitative research performed among students from all study years would offer informative data.

## 5. Conclusions

The results confirm that the curricular and extracurricular activities performed in the Faculty of Medicine from Cluj-Napoca, Romania, stimulated positive attitudes of undergraduate medical students toward research, as well as their motivation and interest into this field, and for some of them also caused possible long-lasting effects relating to their skills and involvement in some forms of research. This interest is fueled, also, by the formal rules introduced by the University that demand that each student prepare and present a thesis in order to complete their studies.

Moreover, the data offer information which could guide further actions needed to improve this field. As studies from other countries also underlined, several measures might help in stimulating translation of positive attitude and interest into real actions and skill development [1,2,4,5,21]. First, the core curriculum must ensure that relevant and appropriate research expertise is attained by all graduates, while the integration of specific research skills training within several aspects of the undergraduate medical education will make them understand better the importance and relevance of research to the routine practice of all doctors.

Second, extracurricular research activities should be encouraged through institutional initiatives focused on informing students about research opportunities, funding and acknowledgement of student research success, as well as creating opportunities for bringing research-active staff and research-enthusiastic undergraduates together to explore possibilities of cooperation.

Third, motivation of teaching staff to mentor research activities of students are needed, while the establishment of research support groups/offices for students might offer valuable help for stimulating students participation in extracurricular research, as proved by examples from other countries [1,2,5,21].

Last, but not least, this study grounded the framework for developing research for monitoring, developing, and evaluating strategies and tools in order to stimulate involvement, perseverance and obtaining good quality results with regard to research among Romanian medical students, in comparison with tendencies and frameworks developed at European and international level.

## Figures and Tables

**Table 1:** Attitudes regarding research.

Items	3rd Year	5th Year
**Research is important for professional development**		
I totally agree (%) ^a^	33.5	46.4
I partially agree (%) ^b^	56.5	45.2
I do not know (%) ^c^	5.8	5.6
I partially disagree (%) ^d^	4.2	1.6
I totally disagree (%) ^e^	0	1.2
Mean	1.19 *	1.34
**Opinions about factors which influence students’ involvement in research**		
**Personal factors**		
**Personal motivation and interest**		
To very high extent (%) ^f^	60.4	55.6
To high extent (%) ^g^	34.2	39.6
To low extent (%) ^h^	3.4	3.2
Not at all (%) ^i^	0.6	0.4
I do not know (%) ^j^	1.4	1.4
Mean	1.49	1.46
**Desire for improving CV**		
To very high extent (%) ^f^	32.3	29.6
To high extent (%)^g^	47.3	45.6
To low extent (%) ^h^	14.2	18
Not at all (%) ^i^	2.7	4
I do not know (%) ^j^	3.5	2.8
Mean	0.92	0.78
**Social influences**		
**Influences from other colleagues**		
To very high extent (%) ^f^	8.1	19.2
To high extent (%) ^g^	43.6	34
To low extent (%) ^h^	34.7	36
Not at all (%) ^i^	10.8	9.2
I do not know (%) ^j^	2.8	1.6
Mean	0.03	0.18
**Professional influences**		
**Presentations from the teaching staff**		
To very high extent (%) ^f^	40	42.8
To high extent (%) ^g^	43.5	44.4
To low extent (%) ^h^	11.2	8
Not at all (%) ^i^	3.5	2.8
I do not know (%) ^j^	1.8	2
Mean	1.05	1.16
**Help and mentoring from the teaching staff**		
To very high extent (%) ^f^	48.1	55.2
To high extent (%) ^g^	38.5	32
To low extent (%) ^h^	8.8	8.4
Not at all (%) ^i^	2.3	3.2
I do not know (%) ^j^	2.3	1.2
Mean	1.21	1.27
**Institutional factors**		
**Research grants for students**		
To very high extent (%) ^f^	36.9	50.8
To high extent (%) ^g^	49.6	36
To low extent (%) ^h^	8.5	7.6
Not at all (%) ^i^	1.9	2
I do not know (%) ^j^	3.1	3.6
Mean	1.11 *	1.26
**Research training for students**		
To very high extent (%) ^f^	32.3	46.8
To high extent (%) ^g^	48.1	39.2
To low extent (%) ^h^	14.6	11.2
Not at all (%) ^i^	3.1	1.6
I do not know (%) ^j^	1.9	1.2
Mean	0.91 *	1.18

*—statistically significant differences at *t*-test between third- and fifth-year medical students; ^a^—coded as +2; ^b^—coded as +1; ^c^—coded as 0; ^d^—coded as −1; ^e^—coded as −2; ^f^—coded as +2; ^g^—coded as +1; ^h^—coded as −1; ^i^—coded as −2; ^j^—coded as 0.

**Table 2:** Interrelationship between attitudes–results of bivariate correlation analyses ^a^.

	Personal Motivation and Interest	Desire for Improving CV	Influences from Other Colleagues	Presentations from the Teaching Staff	Help and Mentoring from the Teaching Staff	Research Grants for Students	Research Training for Students
**Research is important for professional development**	0.210	NS	0.165	0.220	0.146	0.162	0.172
Individual factors							
**Personal motivation and interest**		0.184	0.245	0.226	0.274	0.244	0.277
**Desire for improving CV**	0.184		0.341	0.181	0.163	0.264	0.164
Social influences							
**Influences from other colleagues**	0.165	0.341		0.298	0.331	0.238	0.267
**Profesional influences**							
**Presentations from the teaching staff**	0.245	0.181	0.298		0.639	0.491	0.453
**Help and mentoring from the teaching staff**	0.146	0.163	0.331	0.639		0.460	0.611
Institutional factors							
**Research grants for students**	0.244	0.264	0.238	0.491	0.460		0.454
**Research training for students**	0.277	0.164	0.267	0.453	0.611	0.454	

^a^—Pearson correlation coefficients which are significant are depicted (*p* < 0.05); NS—non-significant.

**Table 3:** Practices regarding research.

Items	3rd Year	5th Year
**Involvement in research activities**		
No (%)	89.6 *	53.6
Only research related to graduation thesis (%)	1.2 *	28.4
More complex research (%)	9.2 *	18
		
**Participation at medical congress** (%)	81.5	85.2
**Presentations at medical congress** (%)	5.8 *	31.2
**Participation in writing scientific articles** (%)	9 *	25
**Participation in research projects** (%)	8.5 *	21.6
**Research index**		
**0** (%)	16.5	10.8
**1 **(%)	68.5	46.4
**2** (%)	9.6	20.8
**3** (%)	4.2	13.2
4 (%)	1.2	8.8
**Mean**	1.05 **	1.62
**Interest in involvement in research activities during medical studies**		
Yes (%) ^a^	65.4	61.6
No (%) ^b^	8.4	16.8
I do not know (%) ^c^	26.2	21.6
Mean	0.56	0.44
**Interest in involvement in research after medical studies**		
Yes (%) ^a^	67.7	74.4
No (%) ^b^	6.2	6.8
I do not know (%) ^c^	26.1	18.8
Mean	0.61	0.67

*—statistically significant differences at chi 2 test between third- and fifth- year medical students; **—statistically significant differences at t-test between third- and fifth- year medical students; ^a^—coded as +1; ^b^—coded as −1; ^c^—coded as 0.

**Table 4:** Factors associated with involvement in research ^a^.

	Multivariate Linear Regression 3rd Year		Multivariate Linear Regression 5th Year	
Independent Variables ^b^	Standardised Beta (CI) ^c^	R^2^	Standardised Beta (CI) ^c^	R^2^
**Gender**	NS		−0.12 (−0.58–−0.01)	0.01
**Research is important for professional development**	0.17 (0.05–0.29)	0.03	0.17 (0.07–0.44)	0.05
**Personal motivation and interest influence students’ involvement in research**	NS		0.16 (0.05–0.45)	0.02

^a^—Dependent variable–Research index (0-minimum, 4-maximum); ^b^—Independent variables which were included were gender and attitudes regarding research; ^c^—Statistically significant standardized beta are depicted (*p* < 0.05); NS—non-significant.

**Table 5:** Factors associated with interest in involvement in research during medical studies ^a^.

	Multivariate Logistic Regression 3rd Year		Multivariate Logistic Regression 5th Year	
Independent Variables ^b^	OR (CI) ^c^	R^2^	OR (CI) ^c^	R^2^
**Research is important for professional development**	4.10 (2.46–6.83)	0.23	3.85 (2.35–6.29)	0.22
**Presentations from the teaching staff influence students’ involvement in research**	1.55 (1.20–2.01)	0.05	NS	
**Research grants for students influence students’ involvement in research**	NS		1.72 (1.25–2.37)	0.05
**Participation in research projects**	NS		3.68 (1.70–7.96)	0.07

^a^—Dependent variable–Interest in involvement in research activities during medical studies (1–Yes, 0–No/I do not know); ^b^—Independent variables which were included were gender, attitudes and practices regarding research; ^c^—Statistically significant OR are depicted (*p* < 0.05); NS—non-significant.

**Table 6:** Factors associated with interest in involvement in research after medical studies ^a^.

	Multivariate Logistic Regression 3rd Year		Multivariate Logistic Regression 5th Year	
Independent variables ^b^	OR (CI) ^c^	R^2^	OR (CI) ^c^	R^2^
**Research is important for professional development**	1.96 (1.32–2.92)	0.08	2.15 (1.44–3.19)	0.11
**Presentations from the teaching staff influence students’ involvement in research**	1.50 (1.17–1.92)	0.06	NS	
**Help and mentoring from the teaching staff influence students’ involvement in research**	NS		1.40 (1.07–1.83)	0.03
**Participation to medical congress**	2.43 (1.23–4.81)	0.03	2.25 (1.03–4.93)	0.03

^a^—Dependent variable-Interest in involvement in research activities after medical studies (1–Yes, 0–No/I do not know); ^b^—Independent variables which were included were gender, attitudes and practices regarding research; ^c^—Statistically significant OR are depicted (*p* < 0.05); NS—non-significant.

## Data Availability

Data can be obtained on justified cases from the corresponding author.
